# Dataset of received optical power on pork meat for optical in-body communications studies

**DOI:** 10.1016/j.dib.2024.110749

**Published:** 2024-07-15

**Authors:** Syifaul Fuada, Mariella Särestöniemi, Malalgodage Amila Nilantha Perera, Marcos Katz

**Affiliations:** a Centre for Wireless Communications, Faculty of Information Technology and Electrical Engineering, University of Oulu, Oulu 90570, Finland; b Research Unit of Health Sciences and Technology, Faculty of Medicine, University of Oulu, Oulu 90570, Finland

**Keywords:** Near-infrared, Pork meat sample, Optical, In-body communication, Phantoms, Free-space

## Abstract

The utilization of actual biological tissue (e.g., pork meat samples) and tissue-mimicking phantoms for optical-based in-body data and energy transfer studies is crucial. Near-infrared (NIR) light, a part of the light spectrum that falls between visible light and infrared, is highly advantageous as a carrier for data transmission due to its superior ability to penetrate biological tissue, for instance, the human body. Using pork meat samples as a propagation medium for prolonged experiments is challenging due to the deterioration of meat quality caused by drying in the temperature chamber. Typically, a controlled-temperature chamber can be utilized to warm the tissue samples to 37 °C. Some experiments need to be carried out over long periods, in some cases exceeding one hour, including the demonstration of transmitting large-size data (e.g., high-definition images or videos) in real-time through biological tissue using NIR LED. Moreover, for statistical analysis, some experiments need to be repeated, therefore degradation of the tissue sample should be avoided. Furthermore, experiments may also encompass investigations into optical wireless power transfer (OWPT) conducted on biological tissues under NIR illumination and employing energy harvester-based commercial photovoltaic cells (PV) at the receiving ends, which would require a long time to charge the storage (e.g., battery or supercapacitor) fully. Using phantoms for such an experiment is also not straightforward, requiring careful consideration, such as standardization issues. One possible approach to address this challenge is to conduct experiments in a free-space environment (e.g., sample-free) while guaranteeing that the optical power received in free-space is equivalent to that obtained through biological tissue. This can be achieved by carefully controlling the LED's current and arranging the optical channel's distance to achieve comparable results. The received optical power is the primary parameter for comparing free-space and biological tissue setups. This dataset provides settings for NIR LEDs ($P_{max}$ = 375 mW and λ = 810 nm), allowing in-body communication experiments in a free-space environment. The LED's current settings in this dataset (free-space) are equivalent in comparison to those used in a test-bed using biological tissue with 5 (five) different variations of LED currents (i.e., 500 mA, 400 mA, 300 mA, 200 mA, and 100 mA). The dataset consists of six pork meat samples with different thicknesses and fat-muscle layer compositions, resulting in 36 data points. This dataset holds significant potential for reuse in any biomedical research, particularly in the fields of in-body communication and energy transfer utilizing light.

Specifications Table

**Table:** 

Subject	Physical sciences: Optics
Specific subject area	A data-in-brief article is related to optics (i.e., optical wireless communication topic) under the subject of physical sciences.
Type of data	Raw (.xlsx file)
Data collection	This paper presents data from a test-bed for an optical-based in-body communication system, which can be helpful for researchers in this field without the need for biological tissue samples or phantoms in their experimental setup. The data collection was carried out in several steps. We first constructed a test-bed comprising transmitters (NIR LED and LED driver) and receivers (sensor and optical power meter). Experiments were done in two types of propagation medium (i.e., through the biological tissues and on free-space) and conducted sequentially. Initially, different currents were applied to a NIR LED (Thorlabs, ML810NM; $P_{max}$ = 375 mW and λ = 810 nm), i.e., 100 mA, 200 mA, 300 mA, 400 mA, and 500 mA, using an LED driver module (*Thorlabs*, DC2200). Six different samples were used as the propagation mediums, denoted as Samples #1, #2, #3, #4, #5, and #6. The received optical power was measured for each LED current setting using a measurement tool (*Thorlabs*, S121C sensor and PM100D). All samples were heated to 37 °C to match the average human body temperature. Subsequently, we collected the data in a free-space setup. In the free-space experiment, we considered adjusting the optical distance (d) and carefully controlling the current of the NIR LED through an LED driver, aiming to achieve results comparable to those of experimental scenarios previously observed with the tissue samples.
	This dataset provides a test-bed for optical-based in-body communication experiments in a free-space environment under NIR LED, where the LED current settings in the dataset are standardized so that the received optical power remains equal to those used in setups involving biological tissue in each LED current variation (i.e., 100 mA, 200 mA, 300 mA, 400 mA, and 500 mA). The dataset contains measurements taken from six pork meat samples with different thicknesses and compositions of fat and muscle layers, resulting in 36 data points.
Data source location	Experimental design and measurement were performed at the Wireless Medical Communications (WiMec) Research Group, Centre for Wireless Communications, Faculty of ITEE, University of Oulu (TS253, VLC Laboratory, Pentti Kaiteran katu 1, 90,570 Oulu, Finland). The fresh pork meat samples used for the experiment were purchased from a local market near the University of Oulu: Torin Lihamestari (Isokatu 54, 90100 Oulu, Finland).
Data accessibility	Repository name: Dataset of Received Optical Power Measurements on Pork Meat Sample for Optical-Based In-Body Communications Studies Conducted on a Free-Space [1] Data identification number: DOI: 10.17632/f6hwnnw63g.1 Direct URL to data: Mendeley repository (https://data.mendeley.com/datasets/f6hwnnw63g/1)

## 1 Value of the Data

• This data can be used to investigate optical data transmission through the human body under NIR light. The tissue's optical properties (e.g., strong absorption, scattering, and reflectance) challenge data transmission due to these optical properties. The tissue should be warmed to match the average human body temperature, which is around 37 °C [2]; it will pose challenges to maintaining stability in the mentioned temperature level at room temperature for a considerably long-time duration, given that transmitting high-quality images or video streaming through the biological tissue may take several minutes or even hours, particularly in low data rate cases. The risk of tissue overheating can be mitigated by implementing careful heating control, thus preserving tissue health and averting damage from excessive heat. However, challenges may arise if samples get dry (due to the evaporation effect) on their surface for prolonged heating of the tissue sample, leading to changes in the sample's optical properties. Additionally, other problems may arise in this particular situation. To avoid this, free-space data transmission can be demonstrated without any issues. This dataset provides a breakthrough solution by adjusting the LED transmit power to propagate at free-space (sample-free) to be the same as the received optical power level when measuring through the porcine sample.
• This data will also be valuable for OWPT research through the human body under NIR light and PV cells. The efficiency factor of the PV cell when capturing NIR light would be a challenge for researchers who are dealing with it [3], given that the process to charge the in-body device's battery fully may take a few hours (approximately three or more) due to inefficient PV cells for NIR optical wavelengths and very high attenuation due to tissue's properties. At the same time, working with meat samples could pose a problem as the quality of the sample may degrade over time. Free-space readings, which are presented in this dataset paper, can be conducted without any issues rather than using biological tissues.
• The presented dataset is believed to offer valuable data for researchers on simultaneous light information and power transfer (SLIPT) through the biological tissue in both simulation and experimental approaches.

## 2 Background

Optical wireless communications (OWC) have significantly impacted technology trends in biomedical applications for the next decade and beyond [4]. One notable application is the utilization of OWC for in-body communications, which is increasingly being considered as a viable alternative to traditional RF or acoustic technologies [5,6]. Various studies have demonstrated that OWC can offer a low-power solution, mitigate RF radiation and interference issues, serve as an excellent secure and private data transmission approach, and enhance safety for in-body communications [2,7,8].

Due to the early stages of research in this field, extensive trials and experiments are necessary to develop and optimize the in-body communication system exploiting NIR before it can meet clinical application protocols. Typically, these tests employing biological tissue are conducted on humans or animals like mice, pigs, or monkeys *in-vivo*. However, this process is time-consuming and costly and must be approached carefully due to ethical considerations [9]. To address these challenges, researchers can consider using realistic clinical simulation alternatives, which can be based on the use of 1) optical phantoms or 2) *ex-vivo* samples from real pork meat. These two approaches have been widely found in the literature. However, there are obstacles to implementing these approaches. In this context, finding new solutions are required.

The first approach is the use of phantoms. Phantoms are models of the human body or body parts and are useful in biomedical experiments while avoiding any possible harm to animals or humans [10]. These phantoms are created specifically for different tissue types (e.g., general skin, brain, fat, muscle, bone, etc.) and different propagation spectrum types (e.g., microwave, optical, acoustic, optoacoustic, etc.). Advancements in technology, from prototyping to clinical testing, necessitate the establishment of standardized and reproducible protocols for phantom fabrication. This ensures the validation of techniques and reduces variations in the optical properties of the phantoms over time [11]. However, acquiring commercial phantoms can be challenging and expensive, particularly for developing countries, as factors like currency exchange rates, shipping expenses, and taxes significantly impact the research process [12].

The second approach involves utilizing *ex-vivo* methods, typically utilizing pork tissue. However, a challenge arises in obtaining pork in certain countries, particularly those with a large Muslim population [13,14], where researchers may have difficulty finding fresh pork stores or grocery shops that sell it. Additionally, it is essential to adhere to stringent *ex-vivo* experimental protocols. For instance, when using store-bought pork for experiments, it must be heated to a temperature close to the average human body temperature of 37 °C to ensure accurate results [5]; this is one of the crucial issues that should be highlighted when we are dealing with *ex-vivo* experiments as the option for in-body communication studies. Prolonged use of pork samples in experiments at room temperature, such as transmitting big-size data and energy using NIR light through the tissue, can lead to a deterioration in samples quality. Maintaining the temperature of the sample at 37 °C poses risks, such as the tissue getting dry due to excessive evaporation.

The challenges mentioned above are the primary obstacles in experimental optical-based in-body communication. We propose an alternative approach that avoids the need for phantoms or the involvement of pork meat samples, relying on free-space for transmission. By adjusting the emitting power of the NIR LED, the receiver in a sample-free setting can experience nearly identical optical power reception than through a porcine sample at a temperature of 37 °C. Our dataset encompasses the regulation of NIR LED current using an LED driver module and the spacing within the free-space optical channel. Included in our data are parameters such as $P_{max}$ = 375 mW, λ = 810 nm, and various tissue samples with different thicknesses: (a) pure muscle tissue 15 mm, (b) pure fat tissue 15 mm, (c) fatty tissue >20 mm, and muscular tissue >20 mm. This dataset holds significant value for researchers studying in-body communication exploiting light, as it is not currently available in the existing literature.

**Fig. 1:**
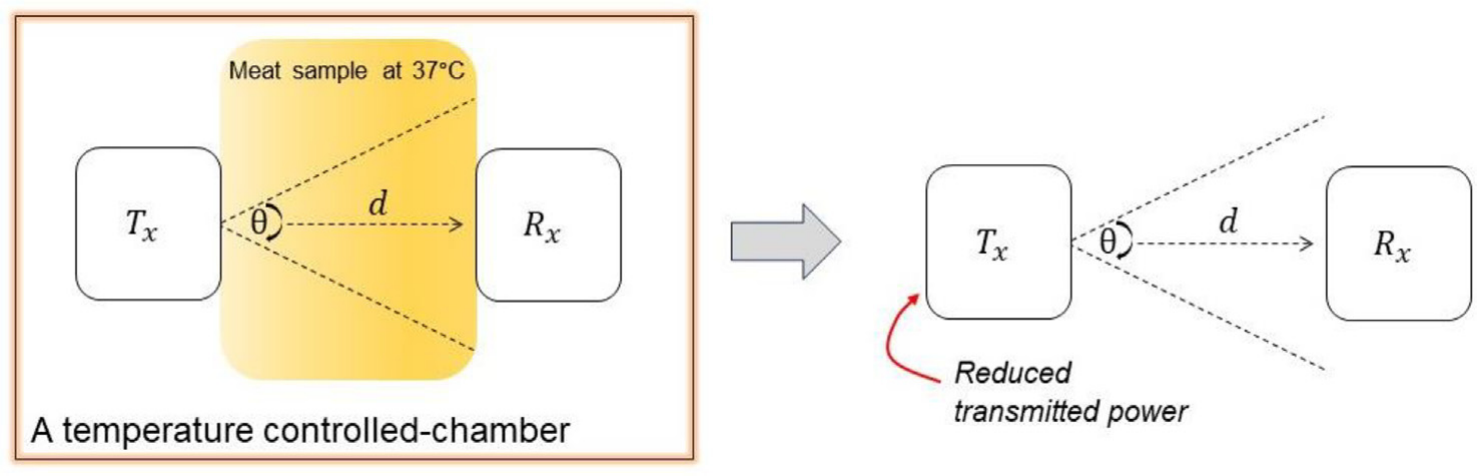
Proposed methodologies for measuring optical power avoiding porcine samples. Note: the system consists of the $T_{x}$ and$\;R_{x}$ where d denotes as the distance between $T_{x}$ and $R_{x}$, and θ denotes the transmitter's field-of-view.

**Contribution**: This paper focuses solely on the necessity of optical power data. For this reason, a simple methodology is proposed, which is considered a novel approach to avoid the deterioration of the porcine samples during the prolonged experiment. This paper provides a useful dataset of optical signals going through *ex-vivo* porcine samples. Fig. 1 shows the illustration of the proposed approach. The left and right sides of Fig. 1 are the conventional approach and the proposed sample-free approach, respectively. As shown in Fig. 1, the conventional methodology is to use the heating chamber to warm up and maintain the sample temperature at the average human body temperature, which is 37 °C. The transmitter ($T_{x}$) and receiver (*Rx*) resided in the heating chamber; this approach has been adopted by the previous study, for instance, in [2].

## 3 Data Description

**Fig. 2:**
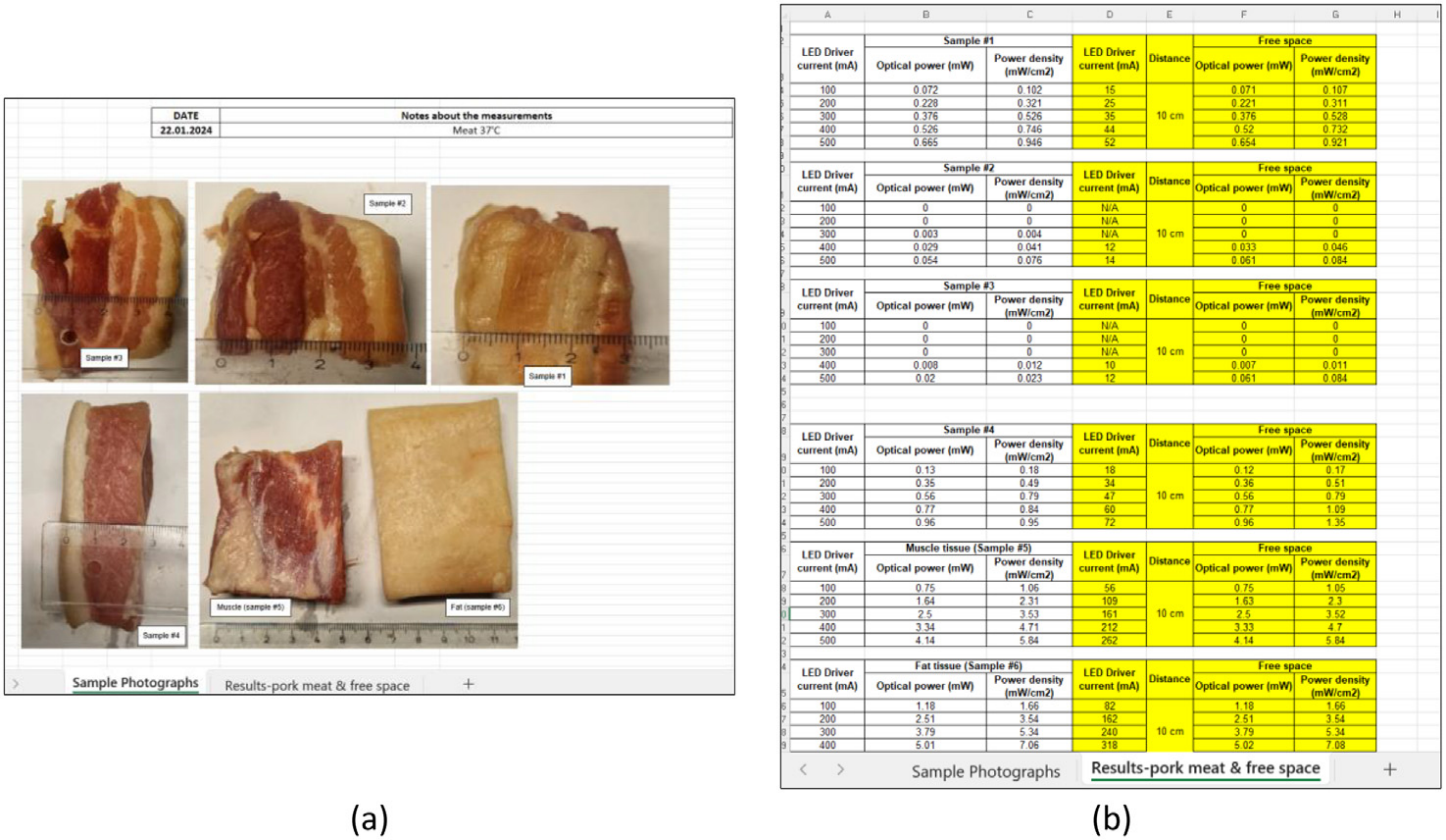
Description of the raw data: (a) sheet 1; (b) sheet 2.

The datasets are contained within a single Excel file with the .xlsx extension, which is accessible in the repository [1]. The file contains two sheets: the first sheet named as “Sample photograph” describes the samples used, as depicted in Fig. 2a), while the second sheet presents the measurements, named “Results-pork meat & free-space”, as depicted in Fig. 2b. The first sheet describes the date of our measurement (January 22, 2024) and sample conditions, based on heated meat at 37 °C.

There are six table measurements in the file (sheet 2). There are two table colors to represent different types of measurements; white indicates the measurements for each sample (#1 to #6), and yellow indicates measurements for free-space. The description table color can be found on sheet 2 at the bottom part. The notation N/A is used to indicate that specific data is not available.

To provide a comprehensive understanding of the data on sheet 2, this section will describe one of the samples. As we have previously discussed, the table is presented in two distinct colors: white and yellow. The white-colored table contains information on LED driver settings (i.e., 100 mA, 200 mA, 300 mA, 400 mA, and 500 mA), sample ID, and measured results of optical power (mW), and power density (mW/cm^2^). The yellow-colored table includes columns for LED power settings, optical distance, and corresponding received optical power and optical power density. The yellow-colored table represents the data for the free-space optical power measurements and serves as a reference for LED current control for the measurements using biological tissue samples. This control will establish a reference point for comparing measurements obtained through biological tissues.

**Fig. 3:**
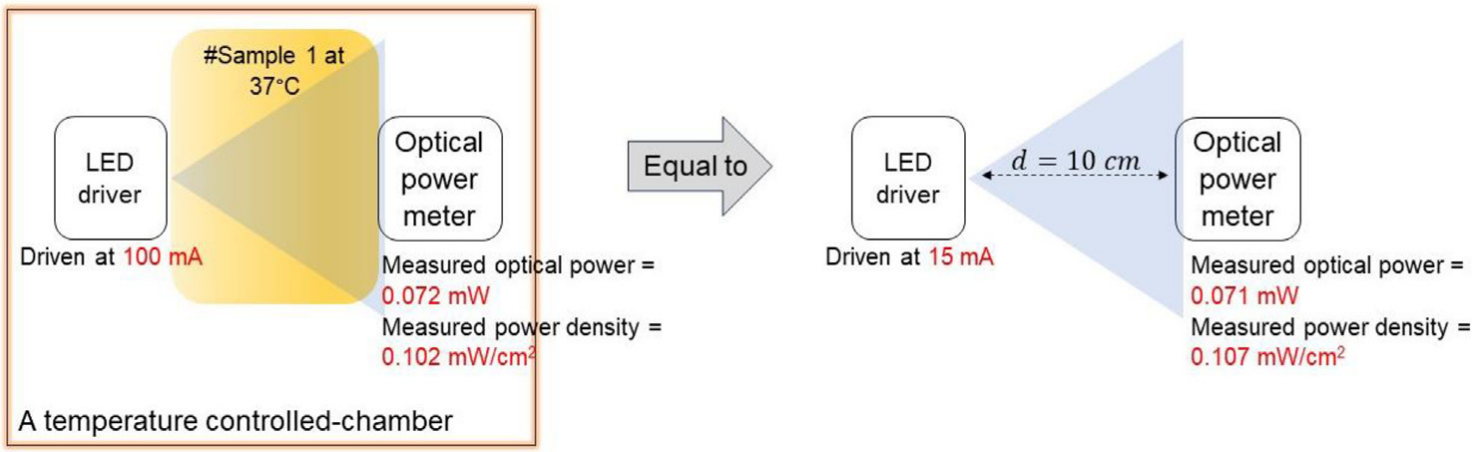
Illustration of the proposed methodologies on #sample 1.

In the case of sample #1, as shown on the white table the measured optical power at 100 mA, 200 mA, 300 mA, 400 mA, and 500 mA is 0.072 mW, 0.228 mW, 0.376 mW, 0.526 mW, and 0.665 mW, respectively. Conversely, when measured using the power density scale, the value is 0.102 mW/cm^2^, 0.321 mW/cm^2^, 0.526 mW/cm^2^, 0.746 mW/cm^2^, and 0.946 mW/cm^2^, respectively. The results indicate a linear relationship between LED driver settings and optical power in a free-space environment (distance at 10 cm). For instance, the optical power and power density at 100 mA in sample #1 are equivalent to those at 15 mA in a 10 cm free-space setting. This pattern is consistent across different current settings, such as at 200 mA in sample #1, corresponding to 25 mA in a 10 cm free space setting. Fig. 3 provides a visual representation of this relationship.

Following this, the table summaries for samples #2, #3, #4, #5, and #6 exhibit similarities to the descriptions provided earlier. As another example, in sample #5, the LED driver current is set at 500 mA, which results in a received optical power of 4.14 mW. In the case of free-space measurements, the transmitted optical power is set at 262 mA at a distance of 10 cm, resulting in the same received optical power as in sample #5, which is also 4.14 mW. Although the measurements taken in the free-space or sample-free setting did not precisely match those obtained from the biological tissue (actual samples), they represent the most optimum value using the available LED driver module by carefully controlling its LED current.

## 4 Experimental Design, Materials and Methods

### 4.1 Preparation

This stage encompassed the preparation of materials and the design of the experimental setup. We used several instruments to implement an optical-based test-bed for in-body communication. The test-bed consisted of a near-infrared LED (*Thorlabs,* M810L3), LED driver (*Thorlabs,* DC2200), a photodetector (*Thorlabs,* PDA36A-EC), an optical sensor (*Thorlabs,* S121C), and an optical power meter (*Thorlabs,* PM100D).

The transmitter side included an LED driver and a mounted NIR LED with a wavelength of 810 nm. The current level of the NIR LED can be manually controlled through the LCD on the LED driver's front panel. The optical power transmitted by the NIR LED was deemed safe for an optical in-body communication system, as it was set according to the ANSI.Z136.1-2007 standard's maximum allowable limit [15,16]. The maximum optical power ($P_{max}$) of NIR LED is 375 mW driven by 500 mA. We varied that current level into 80 % (400 mA), 60 % (300 mA), 40 % (200 mA), and 20 % (100 mA), resulting in 0.3 W, 0.225 W, 0.15 W, and 0.075 W, respectively. The PM100D is a portable optical power and energy meter integrated with an S121C optical sensor with Ø9.5 mm. The gain factor of the mentioned optical power was set to zero, and the input aperture was set to Ø9500 nm to match the sensor's specification. Lastly, we set the bandwidth to “BW Hi,” which means high-bandwidth mode.

### 4.2 Experimental setup

**Fig. 4:**
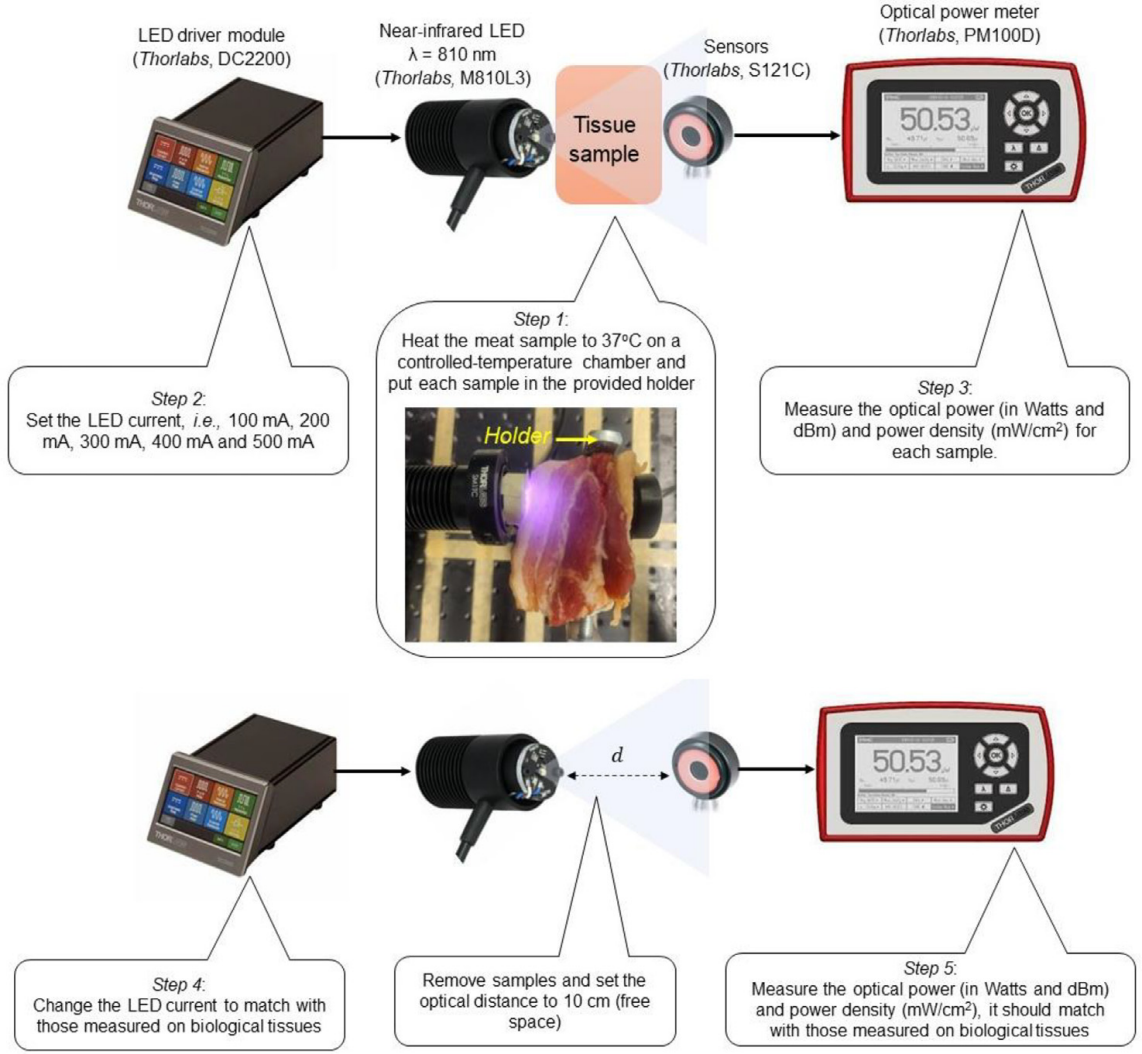
Experimental procedure, containing five steps.

We conducted experiments in a sequential procedure. We first performed measurements on a biological tissue propagation medium and later in free-space after obtaining the data. The visualization of the experimental procedure is depicted in Fig. 4, based on the idea illustrated in Fig. 1.

The detailed description of each step is then elaborated as follows:

### 4.3 Step 1

In the setup, we used biological tissues in the form of pork meat samples. These fresh samples were purchased from a nearby market with an initial temperature of 11 °C (measured using Klein Tools MM400) and subsequently heated to 37 °C, corresponding to the human bodyʼs typical temperature. In this dataset, we provide six samples, which are then denoted as sample #1, sample #2, sample #3, sample #4, sample #5, and sample #6. Before being employed in the experiment, all samples undergo a preliminary heating procedure on a chamber (a plexiglass box equipped with an off-the-shelf heater system), which is located at a different place with the test-bed to protect electronic equipment from the heating process. Once the sample reaches a temperature of 37 °C, it is immediately placed in a designated holder, ensuring appropriate NIR LED and sensor alignment. To mitigate the risks associated with extremely high temperatures, the pork meat samples were carefully maintained at 37 °C and not to exceed 44 °C throughout the heating process, thereby minimizing evaporation and potential harm [17].

### 4.4 Step 2

The NIR LED and sensor were aligned before setting the sample. The tissue sample's surface was illuminated directly by the NIR LED while the sensor was positioned on the opposing end. In this dataset, we limited to five different variations of LED currents controlled by the LED driver module, i.e., 500 mA, 400 mA, 300 mA, 200 mA, and 100 mA [2]. We measured two parameters: optical power (in Watts and dBm) and power density (mW/cm^2^).

### 4.5 Step 3

The measurement of variations of LED current for each sample is then recorded carefully, resulting in 36 data points. These tables are also presented in the dataset [1]. However, it can be seen that in samples #2 and #3, no optical power is received, mainly when the NIR LED is driven by 100 mA and 200 mA, respectively. One possible reason is the excessive thickness of the sample, hindering the effective transmission of power emitted by single-beam NIR LED to the sensor's sensitive area. 150 mW and 75 mW are not enough to penetrate the muscular tissue, with a thickness of around 40 mm. Furthermore, the data also reveals that fatty tissue provides better propagation than muscular tissue. Substantial optical transmission power is necessary to penetrate thick tissues properly while adhering to safety standards to avoid tissue damage. Table 1, Table 2, Table 3, Table 4, Table 5 depict the measurement results on samples #1, #2, #3, #4, #5, and #6, respectively. The thickness of samples #1, #2, and #3 are 30 mm (fatty tissue, where 25 mm fat and 5 mm muscle), 37 mm (muscular tissue, where 12 mm fat and 25 mm muscle), and 40 mm (50 % muscle – 50 % fat tissue), respectively. The thickness of sample #4 is 25 mm; it is composed of 5 mm of fat and 20 mm of muscle. Sample #5 and #6 are pure fat and pure muscle tissues; their thicknesses are 15 mm.

**Table 1:** Measurement results on Sample #1.

Photograph of the sample	LED Driver current (mA)	Sample #1	
		Optical power (mW)	Power density (mW/cm^2^)
	100	0.072	0.102
	200	0.228	0.321
	300	0.376	0.526
	400	0.526	0.746
	500	0.665	0.946

**Table 2:** Measurement results on Sample #2.

Photograph of the sample	LED Driver current (mA)	Sample #2	
		Optical power (mW)	Power density (mW/cm^2^)
	100	0	0
	200	0	0
	300	0.003	0.003
	400	0.029	0.029
	500	0.054	0.054

**Table 3:** Measurement results on Sample #3.

Photograph of the sample	LED Driver current (mA)	Sample #3	
		Optical power (mW)	Power density (mW/cm^2^)
	100	0	0
	200	0	0
	300	0	0
	400	0.008	0.012
	500	0.02	0.023

**Table 4:** Measurement results on Sample #4.

Photograph of the sample	LED Driver current (mA)	Sample #4	
		Optical power (mW)	Power density (mW/cm^2^)
	100	0.13	0.18
	200	0.35	0.49
	300	0.56	0.79
	400	0.77	0.84
	500	0.96	0.95

**Table 5:** Measurement results on Sample #5.

Photograph of the sample	LED Driver current (mA)	Sample #5	
		Optical power (mW)	Power density (mW/cm^2^)
	100	0.75	1.06
	200	1.64	2.31
	300	2.5	3.53
	400	3.34	4.71
	500	4.14	5.84

**Table 6:** Measurement results on Sample #6.

Photograph of the sample	LED Driver current (mA)	Sample #6	
		Optical power (mW)	Power density (mW/cm^2^)
	100	1.18	1.66
	200	2.51	3.54
	300	3.79	5.34
	400	5.01	7.06
	500	6.17	8.68
			

### 4.6 Step 4

**Fig. 5:**
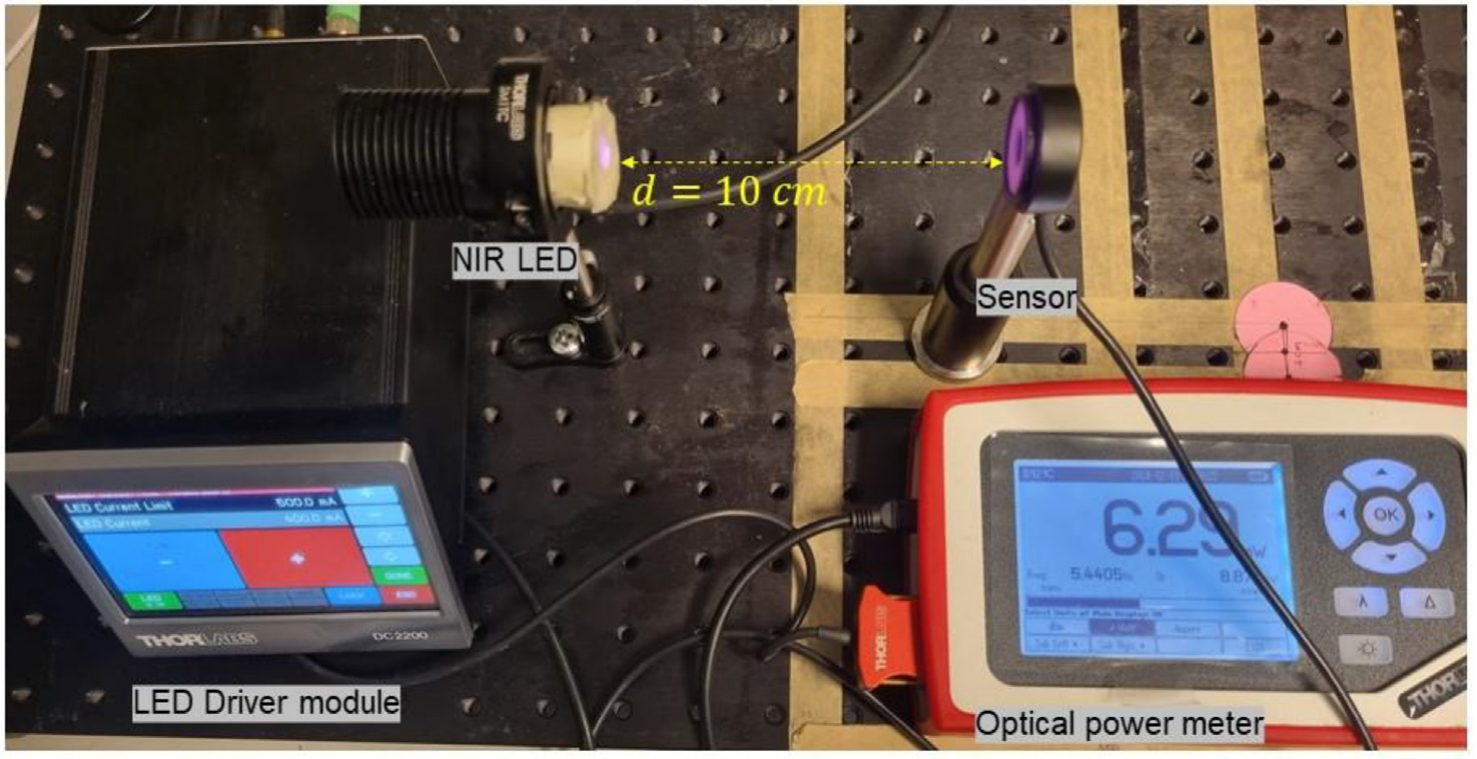
A photograph of the experimental setup on a free-space.

The fourth step is collecting data in a free-space setting (Fig. 5). In this setup, we remove holders and all samples in the propagation medium. The NIR LED light now propagates through an air medium. We considered adjusting the optical distance (d) and carefully controlling the current of the NIR LED through an LED driver, aiming to achieve comparable results to those presented in Table 1, Table 2, Table 3, Table 4, Table 5, Table 6. The most important thing is that the precision of the measurements of received power (mW) and optical density (mW/cm^2^) on free-space should be comparable to those obtained when using LED currents of 500 mA, 400 mA, 300 mA, 200 mA, and 100 mA on biological tissues (as described in *Step 2*). The optical distance was fixed to 10 cm, and afterward, we controlled the LED current through the LED driver module for each sample.

### 4.7 Step 5

Received power (mW) and optical density (mW/cm^2^) on free-space (i.e., sample-free) were successfully obtained, as shown in Table 7, Table 8, Table 9, Table 10, Table 11, Table 12, where this dataset is quite similar to Table 1, Table 2, Table 3, Table 4, Table 5, Table 6. Table 7, Table 8, Table 9, Table 10, Table 11, Table 12 are the measurement results on the proposed setting, which were done without tissue samples in its propagation medium, to correspond to those succesfully measured from Sample #1, #2, #3, #4, #5, and #6, respectively. For example, in Table 6 (using sample #6), the received optical power was 6.17 mW illuminated by NIR LED 375 mW (500 mA), which is equal to results as presented in Table 12 (d = 10 cm) where the measured optical power is 6.17 mW when illuminated by NIR LED driven by 393 mA. As evidenced by Table 5, the received optical power when the NIR LED operates at 200 mA is 1.64 mW, which is comparable to that observed in Table 10 (d = 10 cm) when the NIR LED is driven by 109 mA. In summary, measurements show that the setting in a free-space is quite similar to the biological tissue. The difference between measurements on the pork meat samples and the proposed setting, which was performed on free-space, as assessed through the received power (mW) and optical density (mW/cm^2^), is found to be less than 1 %.

**Table 7:** Measurement results on the proposed setting to correspond to those successfully measured from Sample #1.

Optical Distance (cm)	LED Driver current (mA)	Results on free-space	
		Optical power (mW)	Power density (mW/cm^2^)
10	15	0.071	0.107
	25	0.221	0.311
	35	0.376	0.528
	44	0.520	0.732
	52	0.654	0.921

**Table 8:** Measurement results on the proposed setting to correspond to those successfully measured from Sample #2.

Optical Distance (cm)	LED Driver current (mA)	Results on free-space	
		Optical power (mW)	Power density (mW/cm^2^)
10	N/A	0	0
	N/A	0	0
	N/A	0	0
	12	0.033	0.046
	14	0.061	0.084

**Table 9:** Measurement results on the proposed setting to correspond to those successfully measured from Sample #3.

Optical Distance (cm)	LED Driver current (mA)	Results on free-space	
		Optical power (mW)	Power density (mW/cm^2^)
10	N/A	0	0
	N/A	0	0
	N/A	0	0
	10	0.007	0.011
	12	0.061	0.084

**Table 10:** Measurement results on the proposed setting to correspond to those successfully measured from Sample #4.

Optical distance (cm)	LED Driver current (mA)	Results on free-space	
		Optical power (mW)	Power density (mW/cm^2^)
10	18	0.12	0.17
	34	0.36	0.51
	47	0.56	0.79
	60	0.77	1.09
	72	0.96	1.35

**Table 11:** Measurement results on the proposed setting to correspond to those successfully measured from Sample #5.

Optical distance (cm)	LED Driver current (mA)	Results on free-space	
		Optical power (mW)	Power density (mW/cm^2^)
10	56	0.75	1.05
	109	1.63	2.3
	161	2.5	3.52
	212	3.33	4.7
	262	4.14	5.84

**Table 12:** Measurement results on the proposed setting to correspond to those successfully measured from Sample #6.

Optical Distance (cm)	LED Driver current (mA)	Results on free-space	
		Optical power (mW)	Power density (mW/cm^2^)
10	82	1.18	1.66
	162	2.51	3.54
	240	3.79	5.34
	318	5.02	7.08
	393	6.15	8.68

## Limitations

As mentioned in the value of data section, there are various advantages of this study, such as an optics-based endogenous communication system that may be useful for researchers who do not use biological tissue samples or phantoms in experimental setups. This dataset provides a breakthrough solution by tuning the LED transmit power to propagate in free space and match the received optical power level when measured by the pork meat sample. The presented dataset also provides researchers with valuable data on simultaneous light information and power transfer (SLIPT) through biological tissues, both in simulations and experimental. The experiments were conducted sequentially in two different propagation media (i.e., through biological tissue and in free space). On the other hand, this dataset provides a testbed for optical-based in-body communication experiments in free-space environments under NIR LEDs. It consists of six pork samples of different thicknesses and different fat-muscle layer compositions, where the fresh pork samples used for the experiment were purchased from a local market.

However, this dataset has limitations. The dataset was limited to using NIR LED λ = 810 nm and $P_{max}$ = 375 mW (driven by 500 mA), then conducted strictly on a directional setting where the NIR LED and the sensor are put on the same line. On the actual measurement, it was found that the different angles of both (NIR LED or sensor), the different values of received optical power (the results of measurement data changing as the position of NIR LED/sensor changes). Therefore, it should be considered in future dataset creation. Researchers who will use this dataset should consider the wavelength and optical power of NIR LED issues in their test-beds. This study was carried out solely on the need for optical performance data. Therefore, a simple methodology was implemented (data collection was performed in five steps), which is considered a novel approach as it avoided the degradation of pork samples during the lengthy experiment. This dataset only considers samples of pure fat (15 mm), pure muscle (15 mm), and raw meat consisting of fat–muscle layers. Future work on dataset generation endeavors should take into account more types of porcine samples (e.g., bone and rib), consider higher transmitted optical power to increase the penetration depth to the biological tissues, and explore other wavelengths, such as NIR window I (λ = 700 nm – 900 nm) or II (λ = 1000 nm – 1700 nm). On the other hand, the data should be enriched by variations in angle and direction (e.g., the transmitter or receiver is tilted/shifted from its original position) to represent the misalignment and pointing error issues, which are generally often found in OWC system, including in-body communication cases.

## Ethics Statement

The authors declare that there are no ethical issues regarding this dataset. The authors confirm that their research did not involve human or animal life subjects. Fresh pork meats used in this study were obtained from a local market that sells various types of fresh meat, including porcine. For this reason, it is not classified as an animal experiment.

## CRediT Author Statement

**Syifaul Fuada:** Conceptualization, Methodology, Data curation, Writing – original draft, Methodology, Investigation; **Mariella Särestöniemi:** Formal analysis, Review & editing, Supervision; **Malalgodage Amila Nilantha Perera:** Conceptualization; **Marcos Katz:** Conceptualization, Methodology, Writing – review & editing, Supervision, Acquiring the funding.

## Data Availability

Dataset of Received Optical Power Measurements on Pork Meat Sample for Optical-Based In-Body Communications Studies Conducted on a Free Space (Original data) (Mendeley Data).
